# High‐Definition Optophysical Image Construction Using Mosaics of Pixelated Wrinkles

**DOI:** 10.1002/advs.202002134

**Published:** 2020-10-26

**Authors:** Kitae Kim, Se‐Um Kim, Subi Choi, Kyuyoung Heo, Suk‐kyun Ahn, Jun‐Hee Na

**Affiliations:** ^1^ Department of Convergence System Engineering Chungnam National University Daejeon 34134 Republic of Korea; ^2^ Department of Materials Science and Engineering University of Pennsylvania Philadelphia PA 19104 USA; ^3^ Department of Polymer Science and Engineering Pusan National University Busan 46241 Republic of Korea; ^4^ Reliability Assessment Center Korea Research Institute of Chemical Technology Daejeon 34114 Republic of Korea; ^5^ Department of Electrical, Electronics and Communication Engineering Education Chungnam National University Daejeon 34134 Republic of Korea

**Keywords:** anticounterfeiting, image construction, pixelated wrinkles, reactive mesogens

## Abstract

Despite many efforts in structuring surfaces using mechanical instabilities, the practical application of these structures to advanced devices remains a challenging task due to the limited capability to control the local morphology. A platform that programs the orientation of mechanically anisotropic molecules is demonstrated; thus, the surface wrinkles, promoted by such instabilities, can be patterned in the desired manner. The optics based on a spatial light modulator assembles wrinkle pixels of a notably small dimension over a large area at fast fabrication speed. Furthermore, these pixelated wrinkles can be formed on curved geometries. The pixelated wrinkles can record images, which are naturally invisible, by mapping the gray level to the orientation of wrinkles. They can retrieve those images using the patterned optical phase retardation generated under the crossed polarizers. As a result, it is shown that the pixelated wrinkles enable new applications in optics such as image storage, informative labeling, and anti‐counterfeiting.

## Graphical abstract

A platform that is capable of producing pixelated wrinkles based on programming the orientation of mechanically anisotropic molecules is proposed. The optics based on a spatial light modulator assembles pixelated wrinkles of a notably small dimension over a large area at fast fabrication speed. The patterned birefringence of pixelated wrinkles enables image storage, informative labeling, and anti‐counterfeiting.

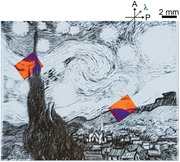

Deformation, as a response to mechanical instabilities, emerges in diverse shapes and a wide range of scales.^[^ ^1^, ^2^ ^]^ Recent advances in manipulating mechanical instabilities have enabled the fabrication of complex, hierarchical topographies.^[^ ^3^, ^4^, ^5^ ^]^ For instance, wrinkles,^[^ ^6^, ^7^, ^8^ ^]^ creases,^[^ ^2^, ^9^, ^10^ ^]^ and folds^[^ ^11^, ^12^, ^13^ ^]^ can be produced in a particular orientation and amplitude. When looking to use these structures in optics,^[^ ^14^, ^15^, ^16^ ^]^ electronics,^[^ ^8^, ^17^, ^18^ ^]^ and other related fields^[^ ^19^, ^20^, ^21^, ^22^ ^]^ as an alternative to classical lithography‐based ones, assembling multidomain structures that spatially vary their orientation and/or morphology is highly desirable. In many cases, the orientation is controlled by the stress distribution during the thermal expansion,^[^ ^7^, ^23^, ^24^ ^]^ osmotic swelling,^[^ ^16^, ^19^, ^25^, ^26^ ^]^ or mechanical deformation.^[^ ^27^, ^28^ ^]^ Multidomain structures are then produced by patterning the stress distribution.^[^ ^29^, ^30^ ^]^ However, these approaches lack the capability to create a large number of individual domains in a small scale and integrate them in a more sophisticated fashion. In the case of using elastic anisotropy, the orientation can be dictated by a principal axis of anisotropy.^[^ ^31^, ^32^, ^33^, ^34^ ^]^ While these approaches afford a large variety of multidomain structures,^[^ ^35^, ^36^, ^37^ ^]^ they are yet to meet high resolution and scalability compared to lithography techniques.

Here, we propose a novel approach to generate pixelated wrinkles that freely change their orientation at a small area (≈0.0064 mm^2^) via controlling the alignment of mechanically anisotropic, reactive mesogens (RMs). A custom‐built optical setup employing a spatial light modulator (SLM) illuminates polarized ultraviolet (UV) light on a particular area of photoalignment layers, such that RMs, when in contact with these photoalignment layers, can reorient to the respective direction of the incident polarization. Wrinkles that are produced during the plasma‐assisted polymerization of RMs can accordingly be aligned. Using a 1280 × 800 array of pixelated wrinkles, our approach assembles complex topographies on various formats of any significant curvatures. We highlight that these topographies show optical patterns based on the birefringence and, as such, permit high‐definition image construction and anti‐counterfeiting.

**Figure** **1**E shows the fabrication process of pixelated wrinkles. We used an azobenzene‐based dye (Brilliant Yellow, Sigma‐Aldrich) as a photoalignment material to spatially regulate the local orientation of wrinkles. The solution of photoalignment materials based on *N*,*N*‐dimethylformamide was spin‐coated on target substrates and annealed to remove the solvent. Upon illumination of UV light, azobenzene dyes in photoalignment layers reorient perpendicular to the incident polarization. The dosage of UV light is precisely controlled to produce the pixelated domain without ambiguous boundaries, which is indispensable to achieve the spatial pixel resolution close to that of patterned UV light (see Figure S2, Supporting Information).

**Figure 1:**
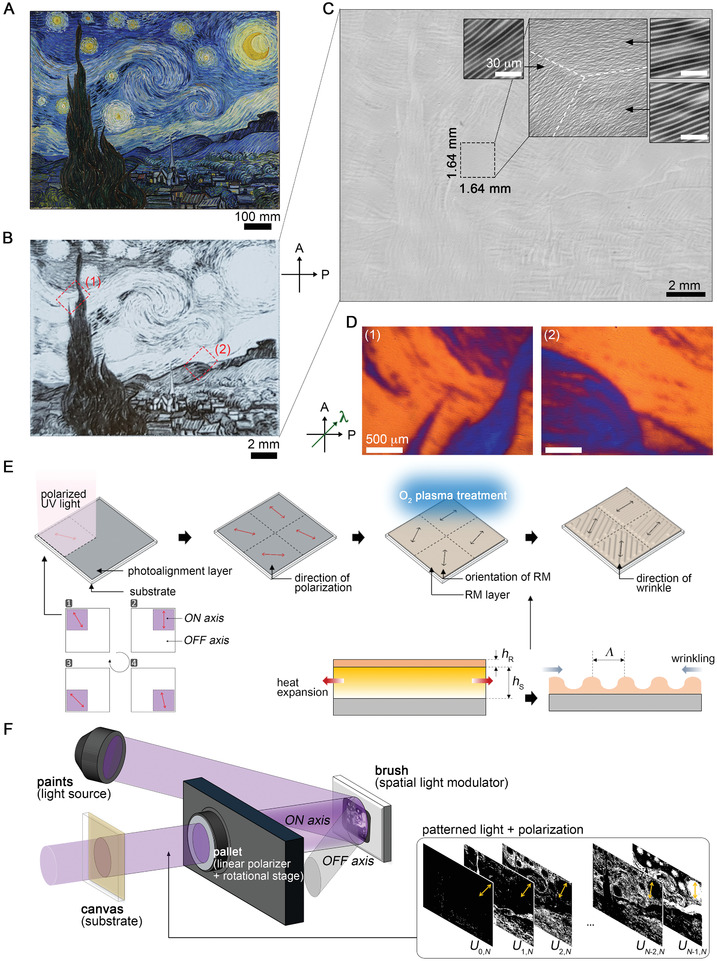
High‐definition, large‐area pixelated wrinkles. A) An original image of the Starry Night painting (921 mm × 737 mm) and B) a reconstructed monochromic image (19 mm × 15 mm) using pixelated wrinkles. Under the crossed polarizers (A and P), the pixelated wrinkle shows different brightness levels due to the variance in the optical phase retardation depending on their orientation. C) The magnified image of pixelated wrinkles. The inset image shows several wrinkle domains of different directions. D) These images are further distinguished by interference color produced from *λ* wave plate. E) Schematic diagram showing the fabrication process of pixelated wrinkles. F) A custom‐built optical setup for photo‐writing the orientation of wrinkles. UV light is patterned by the SLM, polarized, and illuminates the photoalignment layer.

Photoalignment layers are then capable of regulating RMs to align perpendicular to the incident polarization. The RMs are aligned immediately after the deposition process without further annealing process. The polarized optical microscopy (POM) images of the as‐deposited RMs on the UV‐illuminated regions show clear morphology when compared to those on unilluminated regions, confirming that the programmed direction on the photoalignment layer was translated to the RMs (see Figure S2, Supporting Information).

Oxygen plasma treatment promotes the polymerization of RM layers.^[^ ^34^ ^]^ Due to the low penetration of plasma energy, the skin layer of RM is locally polymerized while the underlayer remains unpolymerized. These two layers have different thermal expansion coefficients, and thus when the RM layers are heated from the plasma environment, the polymerized RMs turn into wrinkles by buckling instability. Since typical rod‐like RMs have a higher elastic modulus along with the director (i.e., extraordinary axis) than those perpendicular to the director (i.e., ordinary axes),^[^ ^38^ ^]^ these wrinkles are self‐aligned without controlling the stress distribution. After oxygen plasma treatment, we carried the extra photopolymerization process of the residual RM underlayer using UV light, which produces highly inert, thermally stable wrinkle films. The orientation of wrinkles is maintained regardless of the additional exposure of UV light.

We examined the morphology of irregular wrinkles under several conditions of oxygen plasma treatment time and power (see Figure S3, Supporting Information). At the small level of plasma power (30 W), the amplitude of wrinkles continuously increases with longer treatment time. When the plasma power increases, however, the wrinkles are formed at the early stage of plasma treatment and diminished as further treated. This phenomenon becomes more pronounced as the plasma power increases, implying that the RMs are decomposed at the high plasma power. We optimized the oxygen plasma treatment at the power of 50 W for 1 min, concerning both the throughput and the resolution of wrinkles. The period and the amplitude of wrinkles produced at this condition are set to be ≈4 and ≈1 µm, respectively.

In our optical setup, UV light is first patterned by the SLM, subsequently passes through a linear polarizer that is mounted on a motorized rotation stage, and finally illuminates photoalignment layers (Figure 1F). The mosaics of pixelated wrinkles with multiple orientations are produced by the sequential illumination of patterned UV lights with different angles of the polarization. The concept of image storage relies on using the optical phase retardation of pixelated wrinkles. Wrinkles have a uniaxial birefringence (Δ*n* = *n*_e_ – *n*_o_) with an optical axis along the wrinkle direction. When light propagates through wrinkles placed between the crossed polarizers, the intensity is a function of the orientation angle of wrinkles with respect to the bottom polarizer (*θ*). The intensity can be described as

(1) $$\begin{array}{c} I\;=\frac{1}{2}\;\left(1-\cos\Phi \right)\sin^{2}2\theta \end{array}$$

where *Φ* is the optical phase retardation that is determined by the thickness (*d*) and the birefringence (Δ*n*) of wrinkles at the given incident wavelength (*λ*) (*Φ* = 2*π*Δ*nd*/*λ*). Wrinkles are black and white states when *θ* = 0° and *θ* = 45°, respectively. In the white state, the intensity is maximum irrespective of the magnitude of the optical phase retardation. The value of *θ* for arbitrary gray levels is then placed between 0° and 45°. We define the angular unit (*θ_N_*) for increasing one gray level in *N* gray levels monochromic (*N*G) images as

(2) $$\begin{array}{c} \theta_{N}=\frac{\pi }{4\left(N-1\right)}\; \end{array}$$

Therefore, the angle for *f* gray level in *N*G images is *fθ_N_*. As a representative example, we emulate the Starry Night painting (Figure 1A) using a 2D array of pixelated wrinkles (Figure 1B,C). Microscopic images of these wrinkles show a notable degree of freedom in orientation, with high fidelity in the period and the amplitude (magnified images of Figure 1C). We examine the alignment of molecules at pixelated domains using a *λ* (≈530 nm) wave plate inserted between the crossed polarizers at 45° with respect to the bottom polarizer (Figure 1D). The blue and red interference colors correspond to the orientation of molecules along *θ* = 45° and *θ* = 0°, respectively, and coincide to the orientation of wrinkles.

We study the trends in the transmittance change of both as‐deposited RMs and wrinkles depending on the orientation angle *θ*. The transmittance of as‐deposited RMs at several angles (*θ* = 0°, 9°, 16.5°, 22.9°, 28.5°, 33.5°, 38°, and 45°) agrees with numerical values (0, 0.08, 0.27, 0.49, 0.63, 0.78, 0.87, and 1, respectively, in **Figure** **2**A,C). The transmittance of wrinkles is correlated to that of as‐deposited RMs, but with small deviations that result from the light grating of the wrinkles (Figure 2B,C).

**Figure 2:**
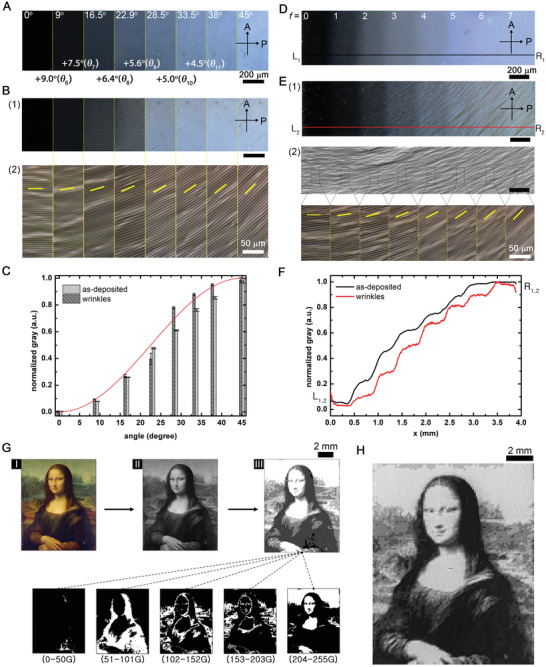
Image construction enabled by the patterned birefringence of wrinkles. A) POM images of the as‐deposited RMs on individual photoalignment layers. RMs are oriented at several angles *θ* (0°, 9°, 16.5°, 22.9°, 28.5°, 33.5°, 38°, and 45°) with respect to the bottom polarizer (P). B) 1) POM images and 2) magnified OM images of wrinkles produced from RMs in (A). Yellow lines in (2) represent the orientation of wrinkles. C) The normalized gray levels of the as‐deposited RMs in (A) and wrinkles in (B) as a function of the angle *θ*. Equation (1) gives the red curve. D) A POM image of the as‐deposited RM that changes the orientation on the single photoalignment layer. The angle *θ* increases by *θ*_8_ (0°, 6.4°, 12.8°, 19.3°, 25.7°, 32.1°, 38.6°, and 45°). E) 1) A POM image and 2) a magnified OM image of the multidomain wrinkle produced from the RM in (D). Yellow lines in (2) represent the orientation of wrinkles. F) The normalized gray levels of the as‐deposited RM in (D) measured from L_1_ to R_1_ and the multidomain wrinkle in (E) measured from L_2_ to R_2_. G) The image construction process using pixelated wrinkles. The original Mona Lisa painting (I) is converted to the monochromic image (II). Pixels of (II) are sorted into several groups according to the gray levels to produce a set of binary maps for patterning UV light. For instance, gray levels ranging from 0 to 50, 51 to 101, 102 to 152, 153 to 203, and 204 to 255 can be categorized into five groups. The mosaic of pixelated wrinkles produced from binary maps reconstructs the monochromic image of II (III). H) The reconstructed 9G image of the Mona Lisa painting. The size of pixels is 110 × 110 µm^2^.

To elucidate the grating characteristics of wrinkles, we examine the diffraction patterns (screen‐sample distance *z* = 37.5 mm) of a He–Ne laser (central wavelength *λ* = 632.8 nm) beam passed through the wrinkles (see Figure S5, Supporting Information). The period of wrinkles (*Λ*) is given as

(3) $$\begin{array}{c} \Lambda \;={\left[\frac{{\left(2\pi \right)}^{6}}{18\left(1-\nu_{R}^{2}\right)}{\left(h_{R}h_{S}\right)}^{3}\frac{E_{R}}{E_{S}}\right]}^{1/6} \end{array}$$

where *ν* is the Poisson's ratio, *h* is the thickness, and *E* is the Young's modulus, and the subscripts R and S represent the rigid skin layer and soft underlayer, respectively.^[^ ^39^ ^]^ At the optimized oxygen plasma treatment condition (50 W for 1 min), we prepared several periods of wrinkles (3.8, 4.5, and 7.4 µm) by varying the value of *h*_R_ *h*_S_ using the spinning rates of 5000, 3000, and 1000 rpm, respectively. We measured the first‐order diffraction angles (*ϕ*_1_) to be 8.7°, 6.0°, and 4.4° at the period of 3.8, 4.5, and 7.4 µm, respectively, which agrees well with the grating equation *ϕ*_1_ = sin^−1^(*λ*/*Λ*) (see Figure S5B, Supporting Information). The total energy of diffraction patterns measured from a photodiode meter (PM100USB, Thorlabs) with sensor of S120VC (Thorlabs) shows a negligible transmittance decrease of wrinkles (98.6%), confirming that the transmittance trend of as‐deposited RMs and wrinkles is quantitatively coincidental (Figure 2C,F). Furthermore, our pixelated wrinkles show a remarkably high tuning range of the period (*Λ* = 3.8–7.4 µm) by simple adjusting the spinning rate,^[^ ^40^, ^41^, ^42^ ^]^ and afford spatially tunable grating domains by varying the orientation of wrinkles for more sophisticated optical functions.^[^ ^43^, ^44^ ^]^

When the multidomain is formed on single surfaces, RMs are continuously aligned at the boundary of adjacent domains for minimizing the elastic energy (Figure 2D,E). As observed from the transmittance, the characteristic length of the continuous boundary is reduced when wrinkles are formed (line profiles of Figure 2D–F). It implies that RMs diffuse and/or drift during the thermal expansion and the polymerization. Note that the total numbers of gray levels depend on the angular resolution. Our approach affords the angular resolution up to 4.5 ° (≈0.08 radians), which corresponds to *θ*_11_. The processing time increases in proportion to the number of gray levels. For instance, the fabrication of 11G images requires 11 illuminations of UV light. However, when the angular resolution increases, our approach is highly scalable without the loss of throughput.

Figure 2G describes the general image construction process using pixelated multidirectional wrinkles. The original image (e.g., Mona Lisa painting) (I) is converted to the monochromic image (II). The distribution of gray levels (0 ≤ *P*(*x*,*y*) ≤ 1) in this monochromic image is sorted into subgroups to comprise a set of binary maps, which is represented as

(4) $$\begin{array}{c} U_{f,N}\;\left(x,y\right)=\left\{\begin{array}{cc} 1 & \;\mathrm{if}\;\;\frac{f}{N}\leq \;P\left(x,y\right)<\frac{f+1}{N}\\ 0 & \mathrm{else} \end{array}\right. \end{array}$$

Being patterned by binary maps U*_f_*_,_ *_N_*, UV lights selectively illuminate the pixels that correspond to *U* = 1. In this process, polarization is synchronized as *fθ_N_*. The mosaic of pixelated wrinkles produced from binary maps then comprises the final image (III).

The spatial resolution of the constructed images is refined when reducing the number of the SLM pixels used for comprising one pixel (see Figure S4B, Supporting Information). The individual pixel width (*w*) shows an excellent linear correlation to the number of the SLM pixels (see Figure S4A, Supporting Information). Therefore, the total pixel number of constructed images can increase up to that of the SLM. In the case of using a magnification optics (×15), our optical setup produces the minimum pixel width (*w*) of 80 µm, corresponding to the minimum pixel area of 0.0064 mm^2^. Without magnifying the illumination area, much higher resolution close to the pixel size of the SLM (≈5.35 µm) can be produced. Furthermore, since all pixels of the same gray level are simultaneously illuminated, the increase of spatial resolution can be carried without increasing processing time.

In the case of reconstructing the Mona Lisa painting, we achieved the pixel resolution of 110 µm in a 10 mm × 15 mm printed image with nine gray levels (Figure 2H). It corresponds to 230 pixels per inch (PPI) and a total of 12 467 pixels (91 × 137) in the entire area. The expression of darker levels is further improved when compared to 5G images (see Figure S4B, Supporting Information).

**Figure** **3** shows the pixelated wrinkles produced on curved surfaces. In this case, photoalignment layers were deposited using the dip‐coating process. The illumination of the patterned UV light on photoalignment layers was carried without any modification of the optical setup. We observe that the aligning direction of wrinkles remains unchanged despite a certain degree of incident angle (*θ*_r_) (Figure 3A). Furthermore, morphologies of wrinkles, including the period and the amplitude, show remarkable consistency between different areas (Figure 3B). The radius of curved surfaces (≈14.2 mm) is far more significant than the period of wrinkles (≈4 µm), so that the variation of optical characteristics of wrinkles (e.g., light diffraction) according to the curvature is negligible. We implement the binary pattern of a world map (i.e., *θ* = 45° and *θ* = 90° for the ocean and the continental patterns, respectively) with the pixel size of 440 µm (Figure 3C). This pattern conformally covered the surface without delamination. The ocean and continental regions become sufficiently distinguishable under the crossed polarizers (Figure 3D). However, light scattering is observed when using unpolarized light (Figure 3E). The POM image at the boundary of two regions and optical microscopy (OM) images showing the orientation of wrinkles at each domain confirm that the morphology of wrinkles is close to that formed on the flat substrate (Figure 3F), suggesting that the scattering is produced from off‐axis incident light due to the curvature.

**Figure 3:**
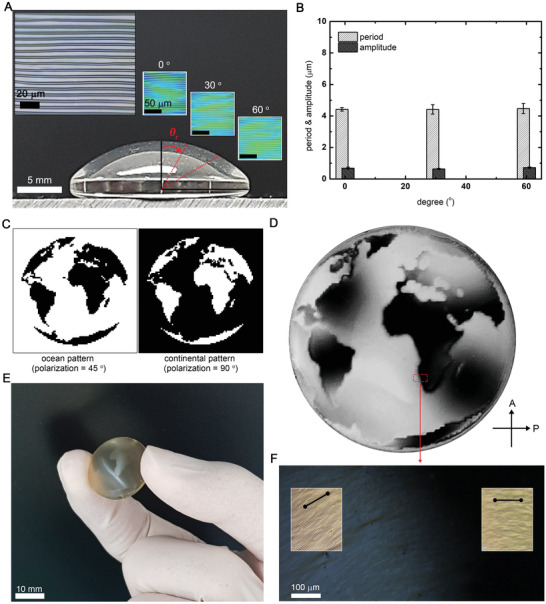
Pixelated wrinkles on curved surfaces. A) A side view of a curved surface, on which the unidirectional wrinkles are formed. The left inset, OM image, is the top view of wrinkles on the center of the curved substrate. The right inset surface profiler images are captured at several radial angles *θ*_r_ (0°, 30°, and 60°) with respect to the axis normal at the center. B) The period and the amplitude of wrinkles at several radial angles *θ*_r_ (0°, 30°, and 60°). C) A set of binary maps designed for patterning a world map. The world map of pixelated wrinkles on the curved substrate observed D) under the crossed polarizers and E) without the crossed polarizers. In (D), the inversion of brightness in some regions is due to the birefringence of the substrate. F) Magnified POM images of the world map at the boundary of two orientation angles. Insets show the OM images with black lines that represent the orientation of wrinkles.

We demonstrate the anti‐counterfeiting concept based on our technology. A quick response (QR) code was constructed using pixelated wrinkles of *θ* = 0° and 45° (**Figure** **4**A) and implemented on a banknote. Under the usual circumstance without the polarization, this QR code is not observed because there are no effective phase differences across the pixels. This image is retrieved only when appropriate optical filters (i.e., the crossed polarizers) are used (Figure 4B,C). The principle of using optical phase retardation can be applicable to UV or infrared light sources, which are currently used to discriminate against counterfeits. This image is also readable when placed on the opaque surface by the reflection mode with one polarizer. We note that other configurations of the optical filter can be employed.^[^ ^45^, ^46^, ^47^ ^]^ The capability of producing pixelated wrinkles with a high pixel resolution and density affords their use in informative labels and security tags to prevent the forgery of products (Figure 4D). The glass bottle used here is a miniature with a radius of 19 mm. The informative labeling based on pixelated wrinkles, being incorporated with the polarized QR code, meets the requirement in the pattern size (>12 × 12 mm^2^) and the image contrast when using a smartphone camera with a polarizing film.

**Figure 4:**
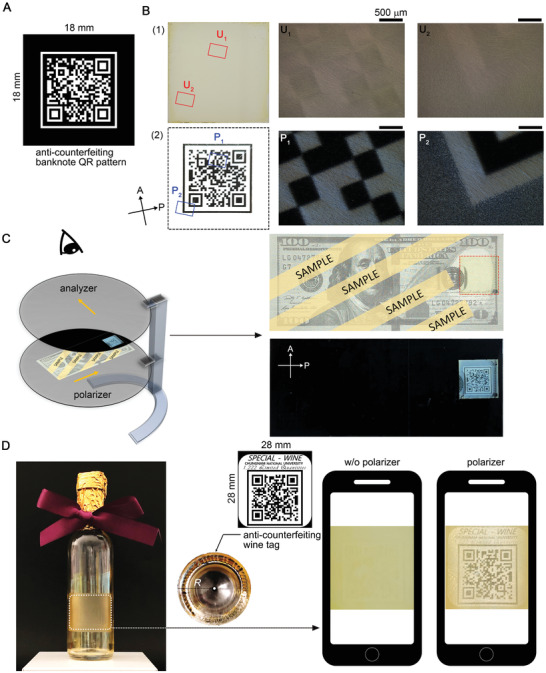
Anti‐counterfeiting and informative labeling using pixelated wrinkles. A) The binary map designed for patterning a QR code. The wrinkles are oriented by *θ* = 45° at the white sector and *θ* = 0° at the black sector. B) Images of the constructed QR code using pixelated wrinkles that are observed 1) without the crossed polarizers and 2) under the crossed polarizers, including magnified images at the boxed regions. C) The schematic illustration showing the concept of counterfeits detection incorporating the pixelated wrinkles. The demonstration shows an example of the anti‐counterfeiting of banknote. D) Informative labeling of pixelated wrinkles. The example shows the labeling on miniature wine bottles for certification. Conceptually, smart devices furnished with a proper optical filter can read this QR code.

The application of these labels at the common class of produces is highly feasible due to freeform shapes and superb scalability without losing the throughput. Implementation of physically unclonable morphologies, which are achieved by mutually using other pixelated wrinkles of arbitrary orientation, may provide a robust solution at higher security‐level applications in a simple way. Instead of using classical bilayer structures in a bulky body, our platform directly records patterns on thin films, collects them in freestanding forms, and relaminates them on different surfaces, allowing more room for practical usages. Our simple, straightforward route for programming the direction of wrinkles achieved high throughput, fast processing speed, and excellent fidelity, and it will bring new possibilities in future studies on structuring surfaces and their advanced applications.

## Experimental Section

##### Preparation of Optical Setup

The optical setup for writing the aligning direction on photoalignment layers comprises a UV light‐emitting diode (365 nm), an SLM (SM7‐405, Sicube Photonics Co., Ltd.), and a motorized rotational stage (K10CR1/M, Thorlabs) furnished with a UV polarizer (LPUV‐100‐MP2, Thorlabs). The SLM is a digital micromirror device that has a wide extended graphics array (WXGA, 1280 × 800 pixels). The polarizer works synchronously with the SLM. Target substrates were mounted on a three‐axis translation stage (DT12XYZ, Thorlabs) to align with the optical setup. A rail (RLA600/M, Thorlabs) and rail carriers (RC4, Thorlabs) were used to adjust the distance between substrates and the optical setup. The intensity of UV light at substrates was controlled to be 2.2 mW cm^–2^. It was measured using a power meter (PM100USB, Thorlabs). The maximum dispersion of the light intensity across the center to the boundary was controlled to be below 7%.

##### Fabrication of Pixelated Wrinkles

In the general process for preparing substrates, quartz slide glasses (HSU‐1000412, Marienfeld) were sonicated in acetone (400Series, Hwashin Tech Co., Ltd.), rinsed by distilled water, dried using a flow of nitrogen (N_2_, 99.95%), and treated by UV‐ozone for 15 min (AC‐6, Ahtech). A 1 wt% solution of an azobenzene‐based dye (brilliant yellow, Sigma‐Aldrich) dissolved in *N*,*N*‐dimethylformamide (99.8%, Sigma‐Aldrich) was spin‐coated on substrates at the rate of 3000 rpm for 30 s and annealed at 60 °C for 1 h. These films act as photoalignment layers. After writing the aligning direction, photoalignment layers were annealed at 90 °C for 10 min to stabilize reoriented molecules. A solution of acrylate‐terminated RMs in propylene glycol monomethyl ether acetate (RMS03‐001C, Merck) was used as an active material to fabricate wrinkles. RMs consist of 4‐(6‐acryloyloxyhexyloxy)‐benzoic acid (4‐cyanophenyl ester), 4‐(3‐acryloyloxypropyloxy)‐benzoic acid 2‐methyl‐1,4‐phenylene ester, 4‐(6‐ acryloyloxyhexyloxy)‐benzoic acid‐(4‐methoxyphenylester), and 2‐methyl‐1,4‐phenylene‐bis[4‐(6‐acyloyloxyhexyloxy)benzoate]. This solution was spin‐coated on photoalignment layers at the rate of 3000 rpm for 30 s. Samples were exposed to oxygen plasma at RF power of 50 W for 1 min (CUTE, Femto Science Co.), where 20 sccm oxygen and 7 sccm argon were used as the reactive gas and the carrier gas, respectively.

##### Imaging Process

Continuous monochromic images were first produced from original images using MATLAB (MathWorks). During the image process, the size and the number of pixels were modified. The pixels in processed images were sorted into subgroups depending on the target gray levels. For instance, when producing 8G images, the pixels of 0–31, 32–63, 64–95, 96–127, 128–159, 160–191, 192–223, and 224–256 gray levels were sorted. Finally, the binary maps were produced based on Equation (4).

##### Characterization

An OM (ECLIPSE Ti2, Nikon) with a 4× or 10× objective lens (S Fluor, Nikon) and a scanning electron microscopy (XE‐100, Park systems) were used to examine the morphology of wrinkles. A large image of high resolution was produced by assembling subimage shots. For instance, the full‐size Starry Night image of pixelated wrinkles (18.5 mm × 15 mm, Figure 1B) was constructed using 9 × 7 subimage shots observed through a 4× objective lens (1608 × 1608 pixels, 1.82 mm per pixel). When assembling subimage shots, each shot was overlapped (30%) with adjacent shots to produce continuous boundary. A laser scanning confocal microscope (Olympus OLS5000) was used to characterize wrinkles formed on the curved surface. The laser was focused on 0°, 30°, and 60° with respect to the apex of the curved surface to compare the period and amplitude of wrinkles created in different locations. The period and amplitude of the wrinkles were measured at three different samples, and average values were reported.

## Author Contributions

K.K. and S.‐U.K. contributed equally to this work. K.K., S.‐U.K., and J.‐H.N. designed the research. K.K., S.‐U.K., S.C., K.H., S.‐K.A., and J.‐H.N. conducted the research and interpreted the results. J.‐H.N. supervised the research and interpreted the results. K.K., S.‐U.K., and J.‐H.N. prepared the manuscript.

## Conflict of Interest

The authors declare no conflict of interest.

## Supporting information

Supporting Information
