# Supplementary material for: High‐Definition Optophysical Image Construction Using Mosaics of Pixelated Wrinkles
Source: Adv Sci (Weinh). 2020 Oct 26;7(24):2002134. doi: 10.1002/advs.202002134 (PMC7740086; doi:10.1002/advs.202002134)
Supplement: Supplementary file 1 — Supporting Information [file ADVS-7-2002134-s001.pdf]

© 2020 Wiley-VCH GmbH

ADVANCED SCIENCE
Open Access

Supporting Information

for *Adv. Sci.*, DOI: 10.1002/advs.202002134

High Definition Optophysical Image Construction Using Mosaics of Pixelated Wrinkles

*Kitae Kim, Se-Um Kim, Subi Choi, Kyuyoung Heo, Suk-kyun Ahn, and Jun-Hee Na**

Supporting Information

High definition optophysical image construction using mosaics of pixelated wrinkles

*Kitae Kim, Se-Um Kim, Subi Choi, Kyuyoung Heo, Suk-kyun Ahn, and Jun-Hee Na**

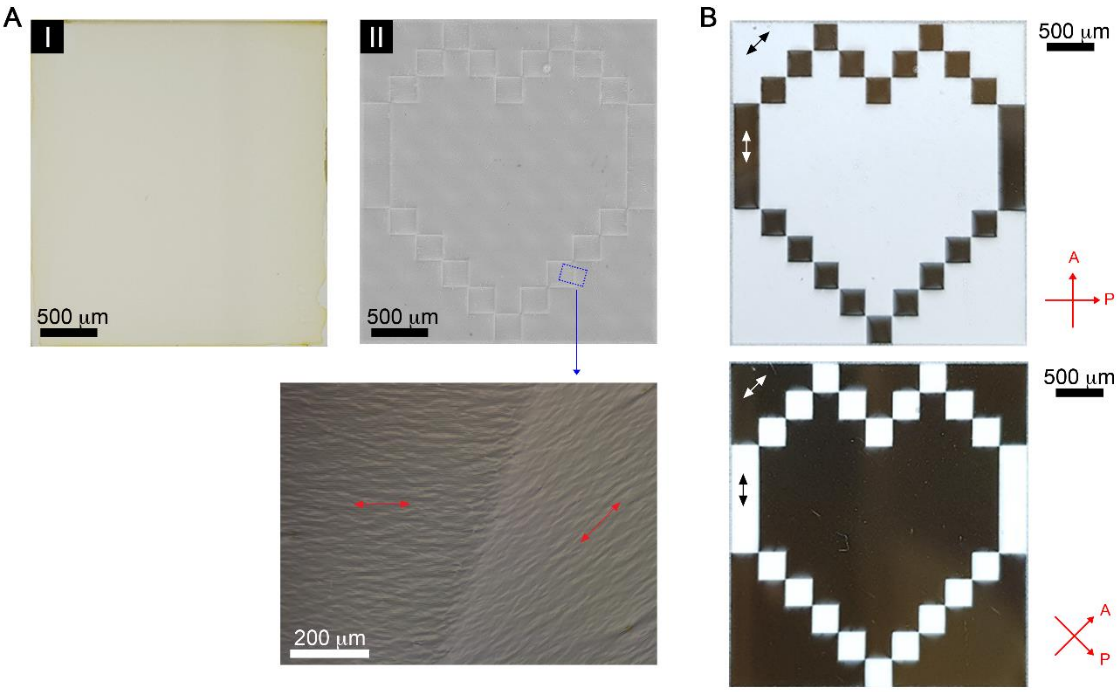

Figure S1. (**A**) Images of (I) the as-deposited RM and (II) the wrinkle, of which direction is patterned to form a heart shape. The magnified image in the blue box shows the boundary between the two wrinkle domains. (**B**) POM images showing the black and white contrast of wrinkles that changes depending on the angle of the crossed polarizers.

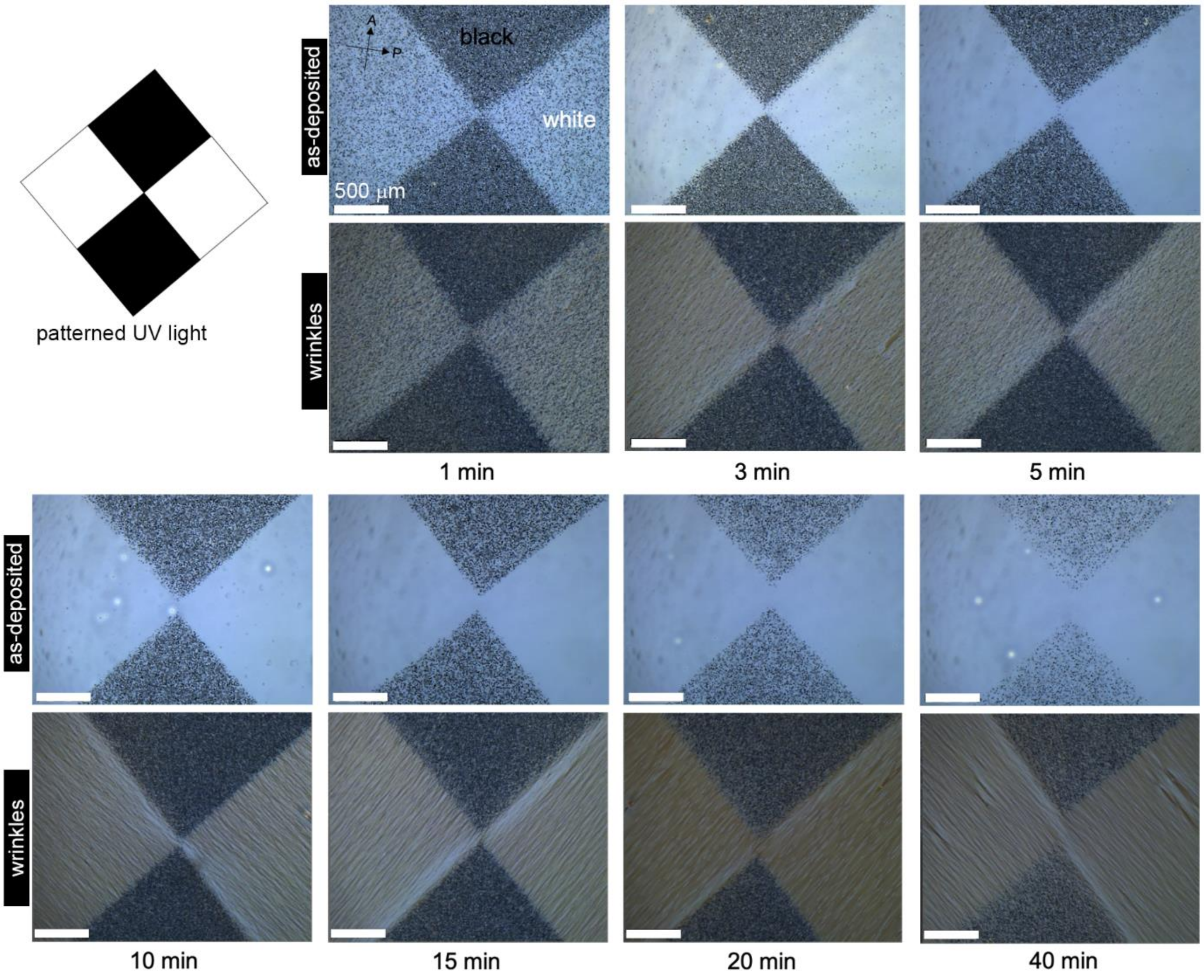

Figure S2. Morphology of as-deposited RMs and wrinkles depending on the illumination time on photoalignment layers. A patterned UV light selectively illuminates a photoalignment layer for several illumination times (1, 3, 5, 10, 15, 20, and 40 min). In the case of short illumination times, the direction of wrinkles is not completely defined due to the weak aligning capability. The boundaries between adjacent domains are blurred when illumination time is longer than 15 min. Therefore, it results in a decrease in the spatial resolution of pixelated wrinkles.

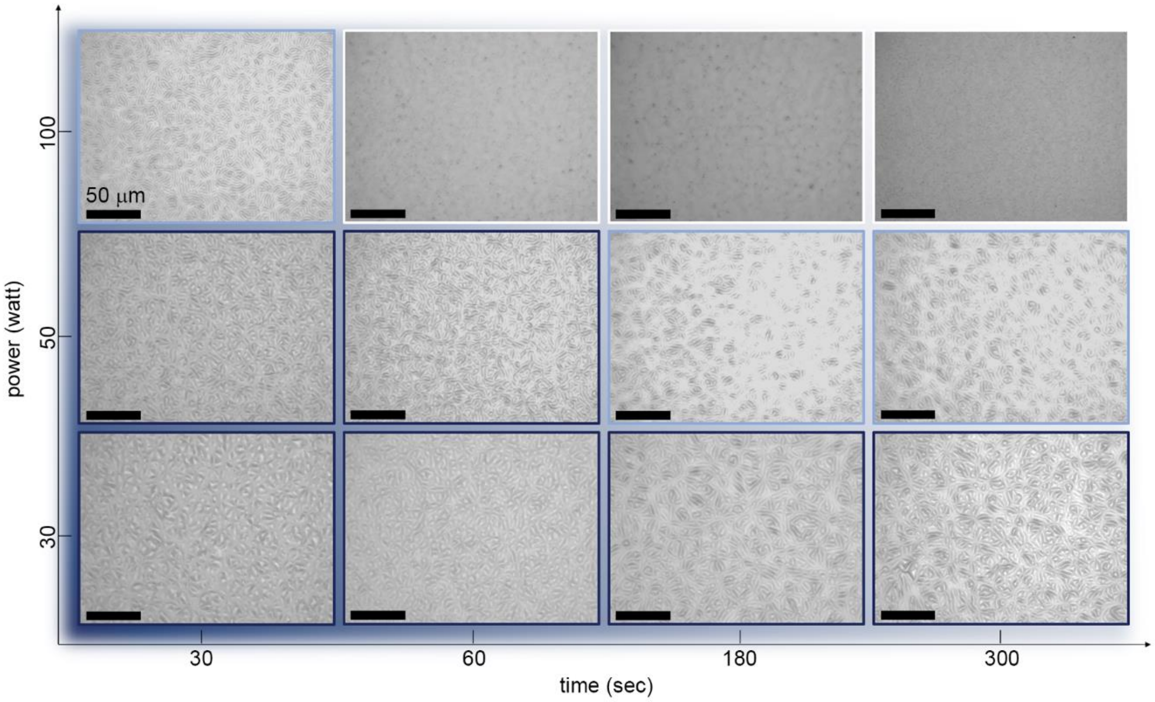

Figure S3. Morphology diagram of wrinkles depending on the oxygen plasma treatment. During the plasma treatment, the formation of wrinkles from the polymerization of the skin layer and the etching process simultaneously occur. At the high plasma power (100 W), the wrinkles are rapidly decomposed as the plasma treatment time increases.

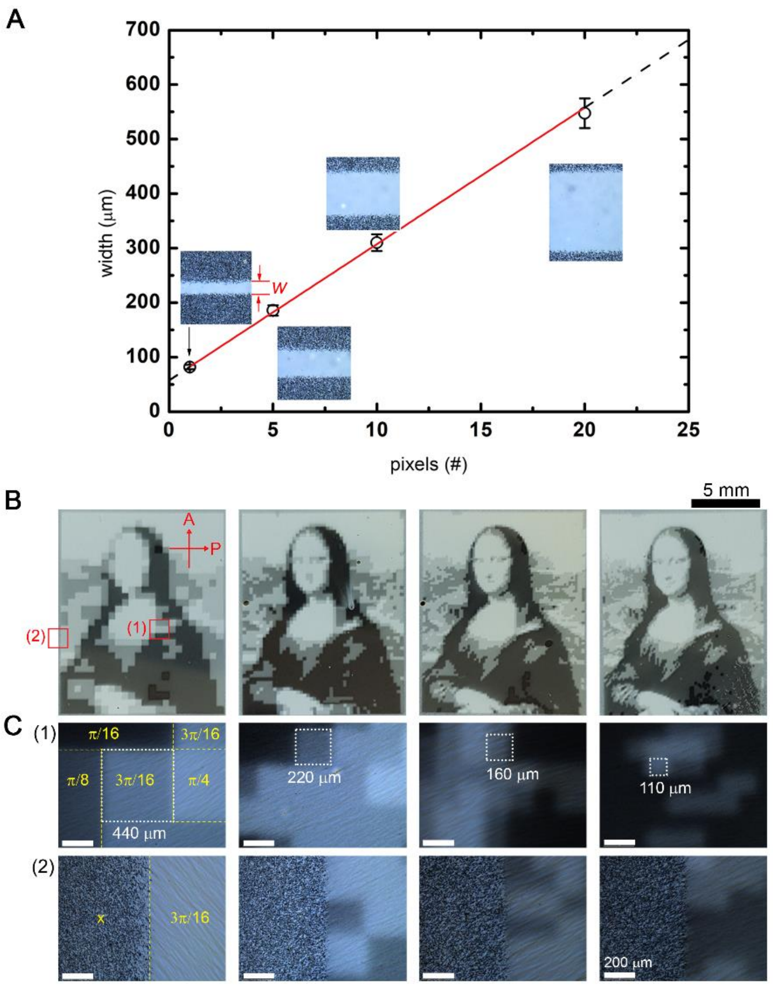

Figure S4. Study on the spatial resolution of pixelated wrinkles. (**A**) The individual pixel width (*w*) of wrinkles as a function of the numbers of the SLM pixels incorporated to construct one pixel when using magnifying optics (×15). (**B**) 5G *Mona Lisa* images of pixelated wrinkles constructed using several spatial resolutions of pixels. From left to right, the resolution is 440 μm, 220 μm, 160 μm, and 110 μm. (**C**) Magnified POM images of the red boxed regions in (b), showing the angles $f\theta_5$ in each domain. In the left domains of magnified images (2), the direction of wrinkles is undefined.

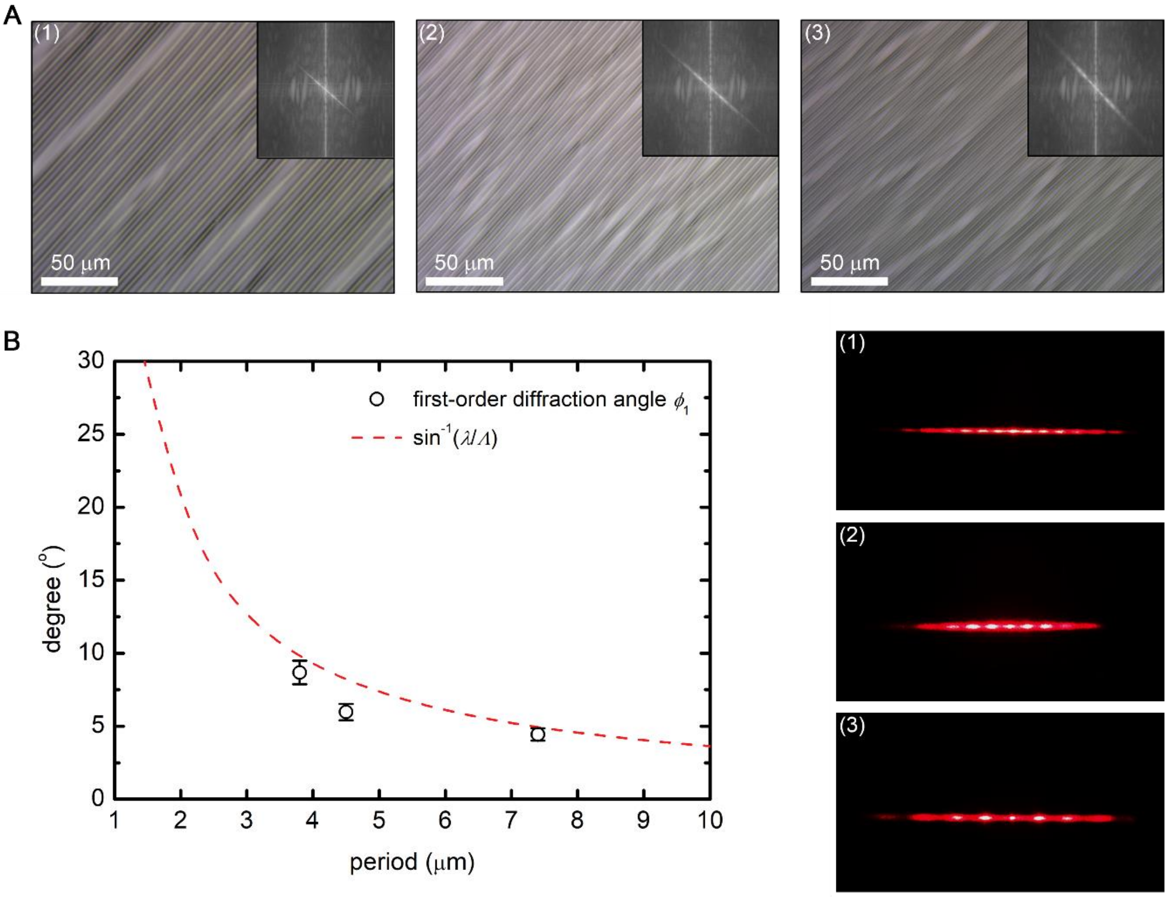

Figure S5. Diffraction grating characteristics of wrinkles. (A) OM images of wrinkles with several periods of 3.8, 4.5, and 7.4 μm. Insets show the fast Fourier transform of wrinkles. (B) The first-order diffraction angles (ϕ_1) which were measured to be 8.7, 6.0, and 4.4 ˚at the period of 3.8, 4.5, and 7.4 μm respectively, which agrees well with the grating equation $\phi_1 = \sin^{-1}(\lambda/\Lambda)$. The diffraction patterns (screen-sample distance z = 37.5 mm) of a He-Ne laser (central wavelength λ = 632.8 nm) beam passed through the wrinkles.
